# Custom-Made Computer-Aided-Design/Computer-Aided-Manufacturing Biphasic Calcium-Phosphate Scaffold for Augmentation of an Atrophic Mandibular Anterior Ridge

**DOI:** 10.1155/2015/941265

**Published:** 2015-05-10

**Authors:** Francesco Guido Mangano, Piero Antonio Zecca, Ric van Noort, Samvel Apresyan, Giovanna Iezzi, Adriano Piattelli, Aldo Macchi, Carlo Mangano

**Affiliations:** ^1^Department of Surgical and Morphological Science, Dental School, University of Insubria, Via Giuseppe Piatti 10, 21100 Varese, Italy; ^2^ITEB Research Centre, University of Insubria, Via Giuseppe Piatti 10, 21100 Varese, Italy; ^3^Academic Unit of Restorative Dentistry, School of Clinical Dentistry, University of Sheffield, 19 Claremont Crescent, Sheffield S10 2TA, UK; ^4^Academic Unit of Prosthodontics, Moscow State University of Medicine and Dentistry, 20 Delegatskaya Street, Moscow 127473, Russia; ^5^Department of Medical, Oral and Biotechnological Sciences, Dental School, G. d'Annunzio University, Via dei Vestini 31, 66100 Chieti, Italy

## Abstract

This report documents the clinical, radiographic, and histologic outcome of a custom-made computer-aided-design/computer-aided-manufactured (CAD/CAM) scaffold used for the alveolar ridge augmentation of a severely atrophic anterior mandible. Computed tomographic (CT) images of an atrophic anterior mandible were acquired and modified into a 3-dimensional (3D) reconstruction model; this was transferred to a CAD program, where a custom-made scaffold was designed. CAM software generated a set of tool-paths for the manufacture of the scaffold on a computer-numerical-control milling machine into the exact shape of the 3D design. A custom-made scaffold was milled from a synthetic micromacroporous biphasic calcium phosphate (BCP) block. The scaffold closely matched the shape of the defect: this helped to reduce the time for the surgery and contributed to good healing. One year later, newly formed and well-integrated bone was clinically available, and two implants (AnyRidge, MegaGen, Gyeongbuk, South Korea) were placed. The histologic samples retrieved from the implant sites revealed compact mature bone undergoing remodelling, marrow spaces, and newly formed trabecular bone surrounded by residual BCP particles. This study demonstrates that custom-made scaffolds can be fabricated by combining CT scans and CAD/CAM techniques. Further studies on a larger sample of patients are needed to confirm these results.

## 1. Introduction

Dental implants are a valid and predictable modality to restore function and aesthetics in completely and partially edentulous patients, with satisfactory high long-term survival rates, particularly in the mandible [1, 2].

Sufficient alveolar bone volume is required to ensure the correct placement of implants and to achieve an aesthetically pleasing outcome [2–4]. However, a variety of processes, including absorption of alveolar bone after tooth loss, periodontal diseases, traumatic injuries, cysts, and tumors, may result in severe alveolar bone defects, with insufficient bone volume to place the implants correctly [5]. In this situation, alveolar ridge augmentation is indicated, either before or in conjunction with implant placement, in order to attain long-term function and an aesthetic outcome [3–5].

Different surgical techniques have been used to overcome alveolar ridge atrophy, including onlay/inlay bone grafting [6–8], guided bone regeneration (GBR) [9, 10], ridge split technique/ridge expansion [11], and distraction osteogenesis [7, 12].

Autogenous bone has always been considered the “gold standard” for alveolar ridge augmentation because of its inherent osteogenic, osteoinductive, and osteoconductive properties [5, 13]. Accordingly, bone reconstructions often involve onlay bone grafts, harvested from either intraoral or extraoral sites [5–7, 13]. However, the use of autografts as onlays has drawbacks, such as additional surgery for harvesting, limited availability, donor site morbidity (which includes risk of infection, bleeding, pain, swelling, and damage to nerves and blood vessels), and high resorption rate of the graft [5, 13]. To overcome these limitations, bone substitute materials such as allografts [8], xenografts [14], and synthetic bone grafts [3, 10, 15] have been introduced.

Whereas all the aforementioned surgical techniques and materials can be successful to augment bone vertically and horizontally, the number of complications and failures of these procedures is still high [4, 5, 13]. Alveolar ridge augmentation remains a major challenge due to anatomical limitations and technical problems, such as the difficulty to shape the bone graft into an appropriate three-dimensional (3D) configuration [5, 13, 16].

Until recently, it was common practice for surgeons to estimate the size and shape of a bone graft on plain radiographs, decide the final shape, and manually cut the scaffold into the desired shape during the operation [16–19]. Unfortunately, this approach is complex and time-consuming and the size and shape of bone graft can be highly inaccurate, as it depends heavily upon the clinicians' ability to contour delicate 3D shapes manually. This may finally result in an unstable clinical outcome [19, 20].

Ideally, bone grafts should be customised to meet individual patient needs, since there are individual variations among patients and differences in damaged parts. The use of grafts that are made to fit precisely according to the 3D shape of the patient's bone defects may improve the vascularization and the biocompatibility of the scaffold following implantation [15–18].

At present, the combination of digital techniques such as model reconstruction based on medical images and computer-aided design/computer-aided manufacturing (CAD/CAM) offers new solutions for planning bone reconstructive surgery in relation to the aesthetic outcomes and the final prosthetic and functional rehabilitation [19–22]. In particular, owing to recent improvements in computer technology combined with advanced computer numerically controlled (CNC) milling units, it is now possible to fabricate 3D custom-made scaffolds in a biocompatible material. A block of bone substitute can be milled into the most appropriate shape that has been preoperatively calculated using 3D simulation [3, 19–24]. This new approach may provide a valuable alternative to conventional procedures that are based on manual intraoperatory modelling of the graft [3, 19, 23, 24].

Until now, however, only a few studies have dealt with custom-made scaffolds for alveolar ridge augmentation [3, 23, 24] and none of these has focused on bone regeneration of the anterior mandible.

The aim of the present report is therefore to document the clinical, radiographic, and histologic outcome of a custom-made, anatomically shaped CAD/CAM scaffold used for the alveolar ridge augmentation of a severely atrophic anterior mandible.

## 2. Case Presentation

An 18-year-old, nonsmoker female patient, with no history of systemic disease, was referred to the Oral Surgery Unit of the Department of Surgical and Morphological Science, University of Varese, Italy, for a fixed implant-supported prosthetic rehabilitation of the anterior mandible. One year earlier the patient had been involved in a car accident and had fractured her anterior symphysis, losing her lower incisors; in that context, internal fixation of the fracture was obtained by means of two rigid plates placed along the upper and lower border of the symphysis.

**Figure 1:**
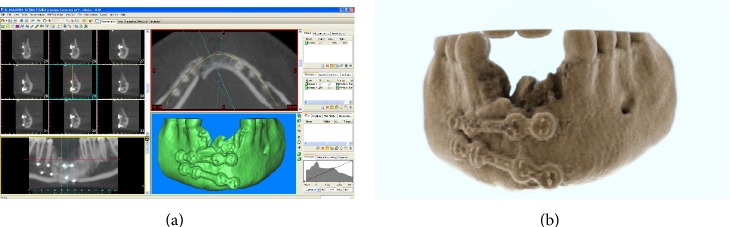
Preoperative situation. (a) 3D reconstruction of the atrophic anterior mandible by means of specific software: a huge vertical bone defect is present in the symphyseal area, combined with a marked reduction in the horizontal alveolar ridge width. (b) Photorealistic rendering of the mandible. The severe posttraumatic atrophy of the mandibular anterior ridge is evidenced.

During the first visit, a complete clinical and radiographic examination was carried out. The patient was wearing a removable partial denture (RPD) as an interim prosthesis to replace the missing mandibular incisors and improve her aesthetic appearance. After removal of the RPD, the first clinical assessment revealed significant contraction of the soft tissues, probably associated with a vertical and horizontal bone defect in the edentulous area, as confirmed by dental casts analysis. Clinical examination revealed unsatisfactory oral hygiene and, consequently, the patient was provided with professional oral hygiene instruction, involving reinforcement in her oral hygiene efforts, followed by a scaling and root planning of the entire dentition. Probing pocket depth (PPD) was measured using a light probing force (of approximately 25 g) with a conventional periodontal probe (PCP-UNC 15, Hu-Friedy Manufacturing, Chicago, IL, USA) at 4 sites per tooth (mesial, midbuccal, distal, and midlingual). The patient was periodontally healthy with PPD values ranging from 3 to 5 mm. Finally, for a better assessment of the bony anatomy, computed tomography (CT) datasets of the mandible were acquired in the Digital Imaging and Communication in Medicine (DICOM) format and immediately transferred to specific segmentation software (Mimics, Materialise, Leuven, Belgium). In this software, the hard tissue threshold was carefully selected so that only bone would be reconstructed from the slices. Accordingly, it was possible to perform an accurate and complete 3D reconstruction of the mandible. Although CT evaluation and 3D reconstruction showed healing of the fracture, they also showed severe posttraumatic atrophy of the mandibular anterior ridge. In detail, a huge vertical bone defect was present in the symphyseal area (9.3, 10.0, 8.6, and 7.8 mm of alveolar bone were lost from the right lateral incisor to the left lateral incisor area, resp.) combined with a marked reduction in the horizontal alveolar ridge width (Figures 1(a)-1(b)).

Given this problematic anatomical situation, the placement of dental implants for supporting a fixed prosthetic rehabilitation was not possible without considering some form of preprosthetic bone reconstructive surgery. Based on the detailed clinical and radiographic examinations, a bone reconstructive procedure with a custom-made synthetic scaffold, followed by delayed implant placement, was proposed to the patient. The patient was fully informed and received a thorough explanation about the planned treatment along with its potential risks and complications. She was also advised about the alternative treatment options of a fixed partial denture on natural teeth or a removable partial denture. After careful consideration, she accepted the proposed treatment and signed a written informed consent form. The study was approved by the Local Ethical Committee at the University of Varese, Italy, and was performed according to the principles outlined in the World Medical Association's Declaration of Helsinki on experimentation involving human subjects, as revised in 2008.

**Figure 2:**
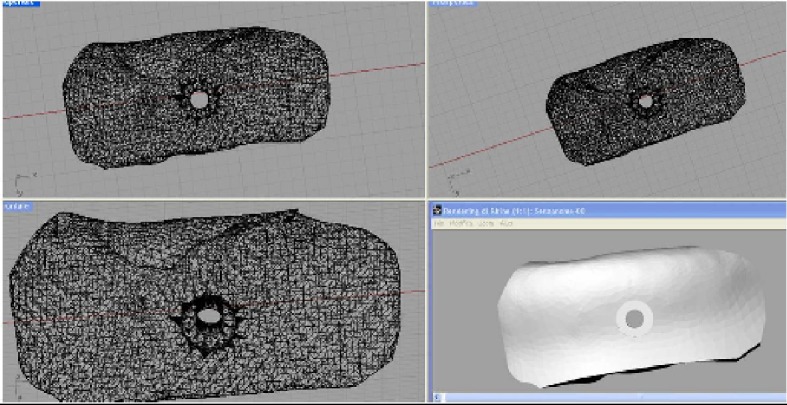
The anatomically shaped, custom-made scaffold is designed. The scaffold includes a hole in its centre to allow the placement of a fixation screw.

One week after the signing of the informed consent form, the process for the fabrication of the anatomically shaped, custom-made hydroxyapatite scaffold started, as previously reported [23, 24]. In brief, the 3D reconstruction of the mandible was transferred as a stereolithographic (STL) file to a 3D CAD program (Rhinoceros, Robert McNeel & Associates, Seattle, WA, USA). With this software, it was possible to reconstruct the alveolar ridge defect virtually and design an anatomically shaped, custom-made scaffold. The anatomically shaped, custom-made scaffold included a hole in its centre to allow the placement of a fixation screw (Figure 2). The 3D geometry of the scaffold was then imported into proprietary CAM software that is used to generate a set of tool-paths for fabrication on a CNC milling machine. A commercially available, synthetic micromacroporous biphasic calcium-phosphate (BCP) block, consisting of 70% beta-tricalcium phosphate and 30% hydroxyapatite (Biocer, Biocer Entwicklungs GmbH, Bayreuth, Germany), was then placed in the CNC milling machine and milled into the exact shape of the 3D project. In this way, an anatomically shaped, custom-made BCP scaffold was manufactured. In addition, a scaffold replica in polytetrafluoroethylene (PTFE) was fabricated; this PTFE replica was intended as a guide for the correct positioning of the hole for the fixation screw (using the BCP scaffold as a guide for drilling the hole could have led to fracture of the fragile scaffold). It took two weeks for the design and fabrication of the BCP scaffold with its replica. The BCP scaffold and its PTFE replica were sterilized before surgery.

**Figure 3:**
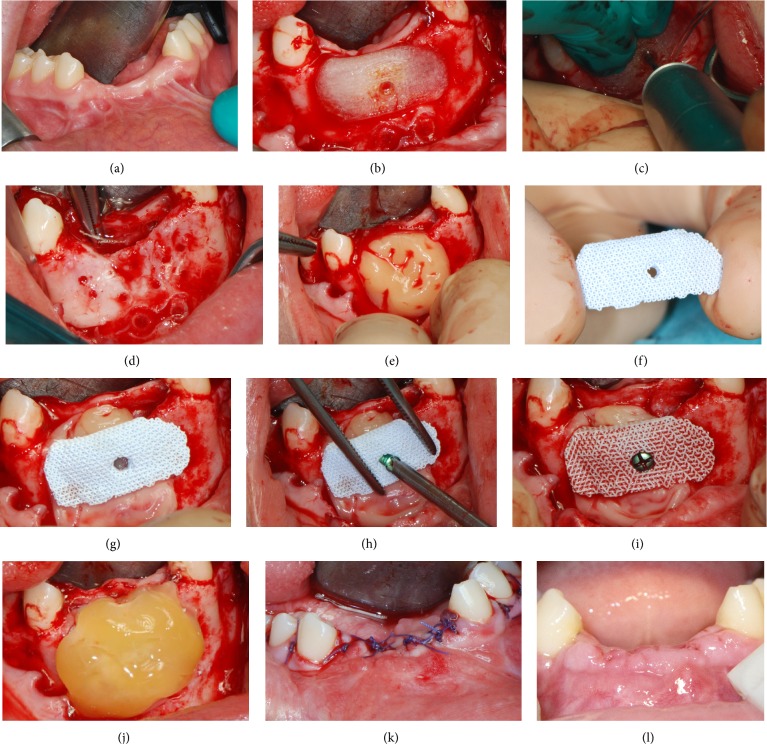
Surgery on patient. (a) Preoperative situation; (b) a PTFE replica is placed in position; (c) the PTFE replica is used for precise positioning of the hole for the fixation screw of the scaffold; (d) multiple small holes are drilled through the remaining alveolar bone into the marrow cavity, with a 1 mm round bur, under copious saline irrigation, to enhance bleeding of the mandibular cortex; (e) a preparation rich in growth factor (PRGF) is prepared and applied to the surgical site; (f) the custom-made BCP scaffold is removed from its sterile packaging; (g) the scaffold is placed in position strictly overlapping the underlying alveolar crest; (h) fixation of the scaffold is obtained by means of a small titanium screw; (i) the BCP scaffold rapidly acts as a sponge, absorbing a large amount of blood from the surgical site; (j) the surgical site is protected with a fibrin membrane; (k) care is taken to obtain a tension-free suture above the scaffold, so as to avoid ischemic damage to the mucosa and suture dehiscence; (l) 2 weeks after surgery sutures are removed.

Two weeks before surgery, the patient underwent periodontal treatment, involving instruction and reinforcement in her oral hygiene efforts, followed by a scaling and root planing of the entire dentition. On the day of surgery the interim removable prosthesis was removed (Figure 3(a)) and local anesthesia was obtained by infiltrating articaine 4% containing 1 : 100.000 adrenaline. Following a crestal incision with two deep lateral incisions, a mucoperiosteal flap was elevated with wide exposure of the mandibular symphysis. The mental neurovascular bundles were identified and protected with a retractor. The fixation plates were unscrewed and removed. Then, the PTFE replica was placed in position and used for precise positioning of the hole for the fixation screw of the scaffold (Figure 3(b)). Once the hole for the fixation screw was precisely drilled (Figure 3(c)), the PTFE replica was removed. Prior to implantation of the BCP scaffold into the alveolar bone defect area, multiple small holes were drilled through the remaining alveolar bone into the marrow cavity, with a 1 mm round bur under copious saline irrigation. This was done to enhance bleeding of the mandibular cortex (Figure 3(d)). A preparation rich in growth factor (PRGF) was prepared, in order to promote healing and tissue regeneration. The preparation was conducted such as to obtain a platelet-rich plasma preparation, a platelet-poor plasma preparation, and a fibrin scaffold. This protocol differed from the original one described by Anitua and colleagues [25] for the lack of sodium citrate and calcium chloride used as anticoagulant and activator, respectively. The platelet-rich plasma preparation was applied to the surgical site (Figure 3(e)). Once the site had been prepared the custom-made BCP scaffold was removed from its sterile packaging (Figure 3(f)) and placed in position, strictly overlapping the underlying alveolar crest and creating a biological rigid fixation (Figure 3(g)). Fixation of the scaffold was obtained by means of a small titanium screw (Figure 3(h)). The BCP scaffold rapidly acted as a sponge, absorbing a large amount of blood from the surgical site (Figure 3(i)). The surgical site was finally covered and protected with a fibrin membrane (Figure 3(j)). During wound closure great care was taken to obtain a tension-free suture above the scaffold, so as to avoid ischemic damage to the mucosa and suture dehiscence (Figure 3(k)). The patient was instructed to avoid hard food and received oral antibiotics, amoxicillin + clavulanic acid 2 g/d for 6 days (Augmentin, GlaxoSmithKline Beecham, Brentford, UK). Postoperative pain was controlled by administering 100 mg of nimesulide (Aulin, Roche Pharmaceutical, Basel, Switzerland) every 12 hours for 2 days, and detailed instruction about oral hygiene was given, along with mouth rinses with 0.12% chlorhexidine (Chlorhexidine, Oral-B, Boston, MA, USA) to be administered daily for 7 days. The patient was seen on a weekly basis during the first 4 weeks. At the first control visit, 7 days after the surgery, a clinically healthy marginal area was present, and no postoperative pain or swelling was reported. There was no bleeding or wound infection. At the second control visit, 14 days after the surgery, sutures were removed (Figure 3(l)). Monthly professional plaque control supplemented this healing phase for 6 months.

**Figure 4:**
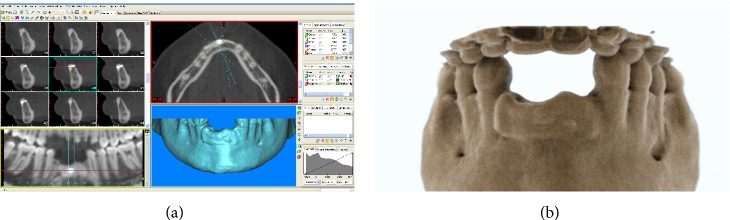
One year after surgery. (a) 3D reconstruction of the mandible by means of specific software: the vertical and horizontal bone gains are clearly evidenced. (b) Photorealistic rendering of the mandible.

**Figure 5:**
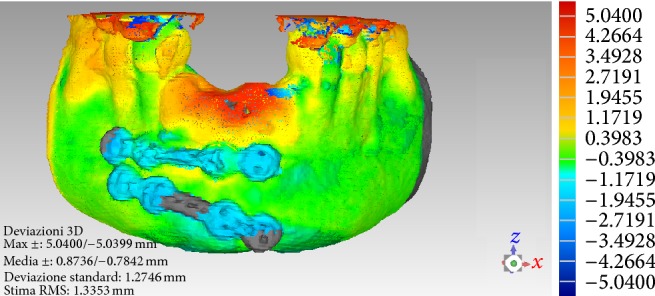
Overlapping of digital images. The DICOM (Digital Imaging and Communication in Medicine) files of the obtained CT datasets, before and 1 year after grafting, are converted into a surface mesh model with digital imaging software (Mimics, Materialise, Leuven, Belgium). The two surface mesh models are then superimposed and rigidly aligned with anatomical landmarks, with the aid of software for the overlapping of digital images (Geomagic Studio, Morrisville, NC, USA). The distance between the 2 surface meshes is presented as color-coded graded figures (blue: tissue loss; yellow/orange/red: tissue apposition; green: little or no modifications) to identify zones of apposition/resorption.

No clinical complications were observed during the 1-year healing period. In this period, the patient wore her RPD as interim prosthesis, primarily for aesthetic reasons. One year after surgery the patient underwent a postoperative CT scan. The new CT datasets were immediately transferred to the 3D reconstruction software (Mimics, Materialise, Leuven, Belgium) for the segmentation of the mandible (Figures 4(a)-4(b)). After that, the vertical and horizontal bone gain was radiographically evaluated by comparing the 3D reconstruction of the preoperative CT scan with that obtained 1 year later, using a method previously described [10, 26]. In brief, data from the preoperative and the postoperative CT scans were segmented using the aforementioned 3D reconstruction software. Based on the result of these segmentations, a surface mesh model was generated according to conventional matching cube algorithms, followed by automated surface mesh model generation. The postoperative mesh model was superimposed on the preoperative mesh model and rigidly aligned by anatomical landmarks with the help of software for the overlapping of digital images (Geomagic Studio, Geomagic, Morrisville, NC, USA). The distance between the 2 surface meshes was presented as color-coded graded figures to identify zones of facial bone resorption. By overlapping digital images the hard tissue gain could be confirmed (Figure 5).

**Figure 6:**
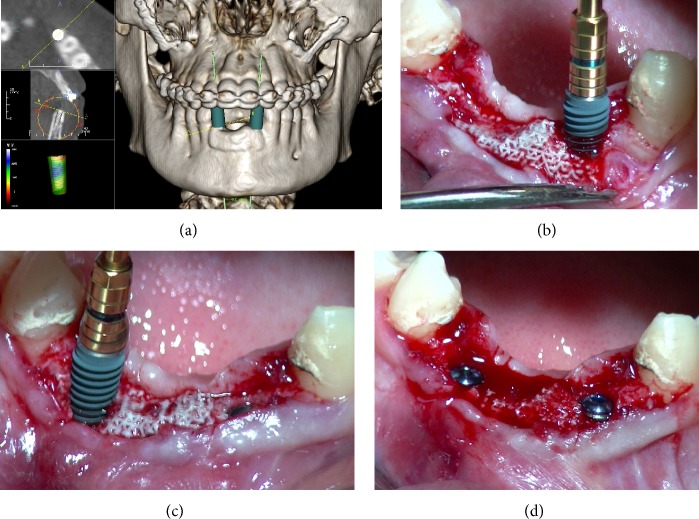
Placement of dental implants in the regenerated area. (a) The placement of two implants is planned with the aim of implant navigation software (Invivo Dental 5, Anatomage, San Jose, CA, USA). (b, c, and d) Two AnyRidge dental implants (AnyRidge, MegaGen Implants Co., Ltd., Gyeongbuk, South Korea), 3.75 mm diameter × 11.5 mm length, are placed in locations numbers 32 and 42.

Since the 3D radiographic examination showed sufficient bone increase and density for implant insertion in the treated anterior mandible, the placement of two implants was digitally planned with the aid of implant navigation software (Invivo Dental 5, Anatomage, San Jose, CA, USA) (Figure 6(a)). Two weeks later, two conical implants with internal connections (AnyRidge, MegaGen Implants Co., Ltd., Gyeongbuk, South Korea) were inserted under local anesthesia by the same surgeon who had performed the grafting procedure. Local anaesthesia was obtained by infiltrating articaine 4% containing 1 : 100.000 adrenaline. A full-thickness crestal incision was made and the soft tissue overlying the reconstructed alveolar process was elevated. The patient showed significant bone augmentation, confirming the possibility of placing two dental implants in the preplanned positions. Accordingly, two implants (3.75 × 11.5 mm) were placed in locations numbers 32 and 42 (Figures 6(b), 6(c), and 6(d)). The threads of the implants used in this study were designed to provide high insertion torque, by increasing their dimensions toward the coronal end of the implant. This specific macrotopographical feature may allow for axial and radial bone compression during implant insertion, and it may be particularly useful in regenerated areas, providing the increased primary stability. An insertion torque of 55 Ncm was registered. Implant stability was determined clinically as the absolute absence of axial or rotational movement by the removal of the implant driver without use of the stabilizing wrench.

**Figure 7:**
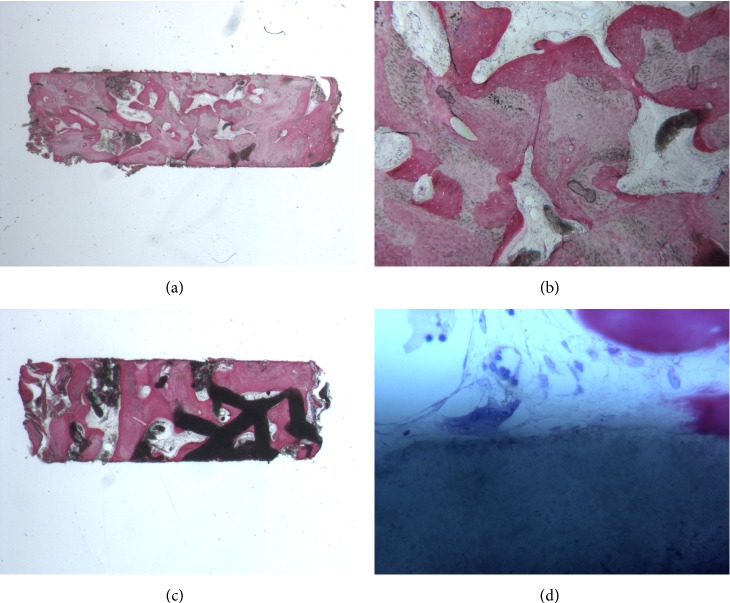
Histological evaluation. (a, b) The left specimen is made of compact mature bone undergoing remodelling, with a few marrow spaces; no residual biomaterial particles are found, as only traces of BCP mixed with mineralized bone matrix are present. (c, d) The right specimen is made of residual particles of BCP surrounded by compact bone. In some areas, traces of residual particles surrounded by mineralized bone matrix are evidenced; multinucleated cells are in close contact with the BCP particles. In the marrow spaces, new blood vessels are evident.

During implant surgery, two bone core biopsies (approximately 2 × 6 mm, one for each site of implant placement) were retrieved with a 2 × 10 mm trephine bur, via a transcrestal path, with the aim of performing a histologic evaluation of the augmented bone. The biopsies were immediately stored in 10% buffered formalin and were subsequently processed (Precise 1 Automated System, Assing, Rome, Italy) to obtain thin ground sections. The specimens were dehydrated in an ascending series of alcohol rinses and embedded in glycolmethacrylate resin (Technovit 7200 VLC, Heraeus Kulzer GmbH & Co., Wehrheim, Germany). After polymerization, the specimens were sectioned lengthwise along the longer axis to about 150 *μ*m using a high-precision diamond disk saw and subsequently ground down to about 30 *μ*m. Two sections were obtained from each specimen. The sections were stained with basic fuchsin and toluidine blue and the histologic evaluation was performed. Histological evaluation revealed compact mature bone undergoing remodelling, marrow spaces and newly formed trabecular bone surrounded by residual BCP particles. The newly formed bone appeared well organized. Close to the porous BCP particles, new bone formation was observed, with newly formed osteoid matrix undergoing mineralization. In detail, the left specimen was made of compact mature bone undergoing remodelling, with a few marrow spaces; no residual biomaterial particles were found, as only traces of BCP mixed with mineralized bone matrix were evidenced (Figures 7(a)-7(b)). The right specimen was made of residual particles of BCP surrounded by compact bone. In some areas, traces of residual particles surrounded by mineralized bone matrix were found; multinucleated cells were in close contact with the BCP particles. In the marrow spaces, new blood vessels were evident (Figures 7(c)-7(d)).

**Figure 8:**
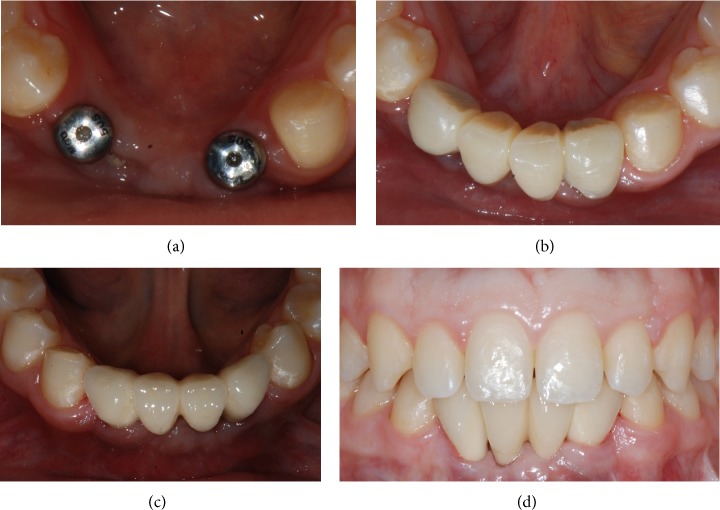
Prosthetic restoration. (a) The implants are left submerged for a period of 3 months, after which they are uncovered and healing abutments are placed. (b) A provisional acrylic resin fixed partial denture is provided. (c, d) Three months later, the definitive ceramometallic restoration is delivered.

The implants were left undisturbed for a period of 3 months after which a provisional acrylic resin fixed partial denture (FPD) was provided (Figures 8(a)-8(b)) which was left* in situ* for a further 3 months. This was replaced with the definitive metal-ceramic restoration (Figures 8(c)-8(d)), which was cemented with a zinc oxide-eugenol cement (Temp-Bond, Kerr, Orange, CA, USA). Occlusion was thoroughly checked. The implant-supported FPD showed good functional and an acceptable aesthetic result.

## 3. Discussion

To perform aesthetic and prosthetic rehabilitation with dental implants, alveolar ridge augmentation is often needed for patients with extensive horizontal and vertical ridge resorption [3–5].

Strategies used to overcome mandibular atrophy include various techniques developed to increase bone volume, such as onlay/inlay bone grafting [6–8], GBR [9, 10], ridge expansion [11], and distraction osteogenesis [7, 12]. Although it has been shown that it is possible to augment bone with all these different techniques, each of these options poses a risk of complications or potential for dimensional graft loss [4, 5, 13, 16]. Moreover, all the aforementioned techniques are based on manual, intraoperative modelling of the graft. This procedure is challenging and time-consuming and may result in an unsatisfactory adaptation of the scaffold to the bony defect [4, 5, 13, 16, 17, 23, 24]. A poor adaptation of the graft material to the recipient site is a major problem during alveolar ridge augmentation, since the lack of mechanical stability of the scaffold may jeopardize the biological response and consequently the treatment outcome [4, 5, 13, 16, 17, 23, 24].

Nowadays, the combination of recent 3D computer simulation techniques, manufacturing technology, and novel bone substitutes with excellent bone tissue conductivity promises to open new interesting horizons for alveolar ridge augmentation. It is now possible to produce an accurate 3D shape of the graft calculated by computer simulation and create a synthetic bone substitute cut exactly into the required shape in a 3D milling machine [23, 24].

In the present report, we describe an onlay technique in the anterior atrophic mandible using a synthetic calcium-phosphate bone graft, shaped with a CAD-CAM system. This approach has the benefits that it avoids the need to harvest autologous bone block and assures a perfect fit of the implant above the alveolar crest. A clinically healthy, young female patient was referred to the Oral Surgical Unit of the University of Varese for treatment with dental implants. The patient presented a severe posttraumatic atrophy of the mandibular anterior ridge, with a huge vertical bone defect in the symphyseal area combined with a marked reduction of the horizontal alveolar ridge width. For this patient ridge augmentation was considered appropriate in order to improve soft and hard tissue volume. In particular, in this clinical situation, a strong rigid graft exceeding 3 mm in height and width was required to allow fixation to the recipient site and 3D stability to withstand muscular force. For these reasons, an onlay technique was selected.

Although autologous bone, harvested from either intraoral or extraoral sites, is currently the most reliable material for alveolar ridge augmentation, with the highest success rate, the use of autografts as onlays has many drawbacks, such as the need for multiple interventions, limited bone availability, the risk of morbidity at the donor site, and high resorption rate of the graft [3, 5–7, 13]. Not to be underestimated, patients prefer a bone substitute block over an autograft block, harvested from an intraoral/extraoral site [3].

Currently, a variety of bone substitute materials, such as allogenic [8], xenogenic [14], or synthetic materials [3, 10, 15], are available for ridge augmentation. An ideal bone substitute should be able to regenerate complex 3D anatomical defects [3, 5, 13, 16, 17, 20, 21]. It should be biocompatible, osteoconductive, and osteoinductive, encouraging appropriate cell differentiation through either soluble or insoluble factor signalling and allowing for delivery of pluripotent cell types [3, 10, 15, 23, 24, 27–29]. It should be structurally similar to bone, possessing mechanical properties similar to the native structures and allowing for function and load bearing [3, 10, 23, 24, 27–29]. It should be synthetic and should not be derived from human cadavers or animals [30, 31]. Finally, it should be easy to shape into various forms and bioresorbable. For these purposes, 3D porous materials are currently used as bone substitutes [3, 10, 15, 23, 24, 27–29]. The 3D porous structure provides space for new bone formation, supports the proliferation of cells, and maintains their differential function, thus mimicking many roles of the extracellular matrix, and its architecture defines the ultimate shape of the new bone [3, 10, 15, 23, 24, 27–29].

Among bone substitutes, synthetic calcium-phosphates materials has been suggested as being able to meet all these criteria [3, 10, 15, 23, 24]. In the present study, a synthetic micromacroporous biphasic calcium-phosphate (BCP) block, consisting of 70% beta-tricalcium phosphate and 30% hydroxyapatite, was selected as the scaffold material. Biphasic calcium-phosphates have been widely used for hard tissue repair and augmentation in different preclinical [32, 33] and clinical settings [10, 34, 35]. The structure and chemical composition of BCP is very similar to that of the mineral phase of bone [10, 32–35]; it is biocompatible, osteoconductive and possesses osteoinductive properties [32–35]; it possesses appropriate porosity for the diffusion of nutrients and the invasion of vascularity from the surrounding tissue and surface chemistry to allow cells to adhere and express the osteogenic phenotype [32–35]. It is characterised by appropriate mechanical properties; it is synthetic and cost-effective, is able to form a suitable shape easily, and ultimately replaces the bone within a short period [10, 32–35].

In the present study, a new protocol for computer-assisted surgery is introduced. This protocol can be divided into four phases: (1) the data acquisition phase, which includes CT scan of the patient; (2) the planning phase, which includes the importing of CT data into a software program for virtual planning and design of the anatomically shaped, custom-made scaffold; (3) the manufacture of the custom-made scaffold using CAD/CAM technology and proprietary CNC milling machine; and (4) the surgical phase, which includes utilizing CAD/CAM-derived scaffold for alveolar ridge augmentation of the anterior mandible. This combination of digital technologies has led to the fabrication of a customized scaffold of greater quality than what could be achieved with manual systems; the scaffold perfectly fitted the recipient site without any amendment required during surgery.

The present protocol offers several benefits. Most significantly it simplifies the surgery, considerably as well as reducing the treatment time [4, 23, 24]. The anatomically shaped, custom-made scaffolds arrive in the operating environment in sterile packaging and only need to be positioned and fixed to the recipient point in the final step of the surgery. The CNC milling process is highly precise, as it offers an extremely accurate, anatomically fitting scaffold, with the benefit of increased stability and excellent reproduction of the patient's bony contour [4, 19, 23, 24]. Improving the precision in adapting the graft is critical to its integration with the surrounding bone: a valid interface between graft and the osteogenic cell lines, and the mechanical stability of the scaffold is needed for new bone formation [4, 16–19, 21, 23, 24]. In the present report, we had the opportunity to use an anatomically shaped, custom-made CAD/CAM scaffold that perfectly fitted the recipient site, without any amendment required during surgery. This precision may have supported the biological integration of the scaffold, resulting in excellent clinical and histological outcomes. The treatment time is considerably reduced, with clear benefits for the patient: in fact, intraoperative time is not consumed by repeatedly modelling the scaffold to the native bone (as in conventional procedures) [4, 23, 24]. The procedure allows a more rapid closure of the surgical wound, avoiding possible sources of contamination of the graft and reducing postoperative discomforts such as swelling and pain, which derive from long and difficult surgical procedures [4]. A consequence of this is that the entire procedure is simplified and more accessible even to less experienced surgeons [4, 23, 24].

Nevertheless, the protocol introduced in this study has some limitations. The first limitation is dimensional and is related to the maximum size of the customized scaffold (12 mm height × 10 mm width). The development of custom-made scaffolds of large dimensions remains challenging because of the requirements for appropriate oxygen and nutrient diffusion throughout the entire construct [4, 23, 24, 36]. To obtain favorable reconstructive results, the bone grafting procedure needs suitable vascular support [36, 37]. Because the osteoblasts require high oxygen tension for bone matrix production, the higher the permeability of the graft to the vascular network, the more effective the new bone formation [36, 37]. An adequate vascular invasion of the scaffold has to be considered an important prerequisite for successful bone regeneration [37]. Without complete vascular invasion and angiogenesis new bone formation is not possible and cells, nutrients, and soluble signals (growth factors), that are mandatory for new bone formation, would be missing [4, 23, 24, 36, 37]. If the scaffold is too big, vascular invasion can be poor, and this could finally jeopardize the healing process [4, 23, 24, 36].

In our present study, PRGF was added to the surgical site. The rationale for the use of PRGF stands in the delivery of a cocktail of proteins and growth factors that may promote wound healing and tissue regeneration to the surgical site [25, 38]. As reported in previous studies, PRGF may be effective in delivering many growth factors such as platelet-derived growth factor, transforming growth factor beta, endothelial growth factor, vascular endothelial growth factor, insulin-like growth factor-1, and fibroblast growth factor. All these soluble factors are capable of promoting healing and tissue regeneration [25, 38]. Other potential limitations of the present CAD/CAM technique include movement artifacts during CT scans and artifacts from filled teeth or metallic restorations close to the edentulous area [4, 23, 24]. In fact, if the patient moves during the radiologic exam, CT datasets can be rather inaccurate and the presence of metallic artifacts may complicate the CAD process and the custom-made scaffold design.

Finally, time is another limitation of this technique. In fact, the entire procedure (from CT scan to surgery on patient) should be carried out in a few weeks, in order to avoid that bone remodelling processes may alter the patient's anatomy; in fact, alteration of the residual anatomy may result in inaccuracy of the custom-made scaffold during surgery. In addition, the amount of time saved by using the CAD/CAM approach is still controversial since whereas the surgical time is considerably reduced, more time has to be spent during the virtual planning and design of the custom-made scaffold [18].

## 4. Conclusions

Digital technology is advancing rapidly in dentistry. Computers are making previously manual tasks easier, faster, cheaper, and more predictable. Personalized therapy is an emerging practice offering tailored solutions to each individual. This approach is envisioned to revolutionize healthcare, through greater cost-effectiveness, efficiency, and improved patient outcomes. In this paper, the authors have described a new digital approach for alveolar ridge augmentation, represented by the use of a CAD/CAM custom-made, anatomically shaped scaffold of BCP, a biomimetic and biocompatible material with the same chemical composition as the bone mineral phase and characterised by high porosity. Despite its limitations, the proposed protocol for alveolar ridge augmentation using CAD/CAM to fabricate custom-made scaffolds plates may represent a viable method of reproducing the patient's anatomical contour, giving the surgeon better procedural control and reducing theatre time. In fact, this technique allowed the successful development of a patient-specific scaffold from a CAD model of an alveolar bone defect obtained from CT images. The benefit is to shorten operating time, improve recovery, and achieve lower morbidity rate. Further clinical studies with longer dental implant follow-up are needed to verify these findings. In the future, the emergence of new rapid prototyping technologies for producing 3D constructs may help to modify the design of the synthetic onlays by adding geometrical features that would facilitate and enhance blood perfusion within the graft; this should improve the bone growth in these onlays* in vivo*. In addition, custom-made scaffolds may be preseeded with cells prior to implantation. The availability of personalized bone grafts engineered from the patient's own stem cells would probably revolutionize the way we currently treat these defects.
